# Obtaining preference scores for an abbreviated self-completion version of the Teen-Addiction Severity Index (ASC T-ASI) to value therapy outcomes of systemic family interventions: a discrete choice experiment

**DOI:** 10.1007/s10198-023-01633-3

**Published:** 2023-09-27

**Authors:** Saskia Schawo, Renske Hoefman, Vivian Reckers-Droog, Liesbet Lawerman-van de Wetering, Yifrah Kaminer, Werner Brouwer, Leona Hakkaart-van Roijen

**Affiliations:** 1 Erasmus School of Health Policy and Management, Erasmus University Rotterdam, P.O. Box 1738, 3000 DR Rotterdam, The Netherlands; 2 University of Connecticut School of Medicine, University of Connecticut, Farmington, USA

**Keywords:** Adolescent, Substance abuse, Delinquency, Mental health, Economic evaluation, Preference-based measure, Discrete choice experiment, Preference scores, C25, I18, I19, H51, H53

## Abstract

**Background:**

Systemic family interventions for adolescents with problems of substance use and/or delinquency are increasingly focused subject of economic evaluations. Treatment effects go beyond improvements in commonly measured health-related quality of life (HRQOL). The Teen-Addiction Severity Index (T-ASI) was identified as capable of capturing these broad outcomes. However, it lacks preference-based scores. An abbreviated self-completion version (ASC T-ASI) was created and validated, covering the T-ASI domains substance use, school, work, family, social relationships, justice, and mental health. This study aimed to obtain societal preference scores for the ASC T-ASI.

**Methods:**

Preferences were elicited in a sample of the Dutch general adult population (*n* = 1500), using a web-based Discrete Choice Experiment. Choice tasks included two unlabeled alternatives with attributes and levels corresponding to the domains and levels of the ASC T-ASI. A pilot study (*n* = 106) informed priors, optimal presentation, and number of choice tasks applied in the main study. Data were analyzed using a mixed multinomial logit model.

**Results:**

Preference scores were logically ordered, with lower scores for worse ASC T-ASI states. Scores were most influenced by reductions in problems concerning the domains substance use, mental health, justice, and family. Tariffs were calculated for each ASC T-ASI state, ranging from 0 (worst situation) to 1 (best situation).

**Conclusions:**

The tariffs enable preference-based assessments of the broad effects of systemic family interventions for adolescents with problems of substance use and/or delinquency. The outcome reflects addiction-related rather than health-related utility and can be used next to generic HRQOL instruments in relevant economic evaluations. Given the source used for the preferences, interpretations and valuation of scores require attention.

**Supplementary Information:**

The online version contains supplementary material available at 10.1007/s10198-023-01633-3.

## Introduction

Economic evaluations in health care often take the form of cost–utility analysis, in which outcomes are captured in terms of quality-adjusted life-years (QALYs) [1, 2], as measured with generic health-related quality of life (HRQOL) instruments like the EQ-5D [3] or SF-6D [4]. This implicitly reflects that many curative health care interventions primarily aim to improve health and longevity of patients. However, in certain health care sectors, the aim of interventions may not (solely or primarily) be to improve health, but to (also) improve broader aspects of quality of life that go beyond health. These broader outcomes may be captured insufficiently by existing generic HRQOL instruments used to calculate QALYs [5, 6]. This issue is gaining attention, for example, in the area of elderly care, where broader measures like ICECAP-O, WOOP, and ASCOT have been developed [7–9]. These measures capture broader life domains than health and are suitable for use in economic evaluations. In other areas, including mental health care and addiction-related treatments, broader preference-based outcome measures are also required but largely lacking [9–14]. The issue of appropriate and comprehensive outcome measures, preference-based, and suitable for use in economic evaluations, is highly relevant in the context of mental health interventions, and particularly for systemic family interventions. These interventions are intended to have broad effects (e.g., related to substance use, family interactions, interaction with peers, and performance at school), that extend beyond the health domain. If not appropriately identified, measured, and valued, such broader effects may fall outside the scope of economic evaluations, risking mis-estimation of the benefits of systemic family interventions. Consequently, the results of economic evaluations may not reflect the actual value for money offered by these interventions and, potentially, result in non-optimal decisions concerning their reimbursement [10, 15, 16]. The results of this study aim to contribute to reducing this risk.

The relevance of this issue is emphasized by the fact that systemic family interventions for adolescents with problems of substance use and/or delinquency are increasingly subject of economic evaluations [17]. However, existing studies are limited in quality and comparability as settings, design, and outcome measures vary extensively [17]. The application of economic evaluations in the field of systemic family interventions is hampered by the lack of preference-based instruments that are validated, sensitive, and feasible to use and that capture all relevant benefits. Systemic family interventions are explicitly directed at improving interactions between the adolescent patient and surrounding systems, and are often used in the context of substance abuse and delinquency [18–20]. Aims of such interventions are diverse and include improvements in family relations, peer interactions, performance at work or school, and reduction of substance use and delinquent activity [19, 21–23]. In a meta-analysis evaluating the effectiveness of outpatient substance abuse treatments for adolescents, systemic family interventions were found to be effective in the treatment of substance abuse [24]. Given that these interventions typically are intensive and costly [15, 21, 26], economic evaluations are important, also to inform reimbursement or funding decisions. This requires validated, broad, multidimensional preference-based instruments that capture the relevant effects of such interventions.

A recent systematic review of the effectiveness literature on systemic family interventions identified existing instruments, which measure relevant benefits beyond health-related quality of life [14]. While no preference-based instruments were found, the Teen-Addiction Severity Index (T-ASI) [27] was identified as a multidimensional instrument capturing the main relevant life domains of adolescents affected by these interventions. Although preference scores for this instrument were lacking, it was considered potentially suitable for adaptation into a preference-based measure for use in economic evaluations of systemic family interventions alongside the use of common HRQOL instruments [14].

The original T-ASI is a relatively long semi-structured interview that measures symptoms of adolescent substance use based on seven domains and five levels of problem severity. The instrument is not a self-report instrument but completed by a therapist together with the patient. Some questions are directed at the patient while others ask the therapist to provide his or her judgment. In order to make the instrument suitable for use in economic evaluations, in which patients commonly report on their own functioning using a self-complete descriptive system, an abbreviated self-completion version of the T-ASI, the ASC T-ASI was created [16; see appendix A and B]. This abbreviated instrument was based on the main patient-reported questions from all domains of the T-ASI, reflecting the functioning of the patient as judged by him or herself. The ASC T-ASI is a broad outcome measure, suitable for self-completion and use in economic evaluations. The ASC T-ASI was subsequently validated, with favorable results [16]. However, since societal preference scores for the ASC T-ASI are lacking, this study set out to obtain such scores, using a discrete choice experiment (DCE).

## Methods

### Sample and data collection

The questionnaire was designed for the purpose of this study and administered to the online panel of Survey Sampling International (now Dynata) in the Netherlands (for more information on characteristics of the Dutch panel see the Panel Book [28]). People who signed up for the panel were invited to participate in this study. Those who accepted the invitation were informed about the purpose of the study and about how their anonymity was guaranteed. They were informed that participation in the study was voluntary and could be stopped at any time, in which case that the data they had provided up to that point would be discarded. By submitting their response at the end of the questionnaire they provided consent for the use of their data for the stated purposes of the study. Participants received no financial compensation. Ethical approval for conducting the study was obtained from the Research Ethics Review Committee of Erasmus School of Health Policy & Management (reference 21-022).

The Dutch translation of the ASC T-ASI [16; Appendix B] formed the basis for the current study and the preference-based measure. In this study, we used a DCE to obtain societal preference weights for all domains and levels of the ASC T-ASI instrument. A professional Dutch translation agency advised us in formulating the instructions of the discrete choice tasks based on reading level B1.

Pilot and main data were collected from the same online panel. As the common source of health state valuations is the general public [1, 2, 39], we elicited preferences for different states described with the ASC T-ASI in a sample representative of the general adult population in the Netherlands in terms of age (18–65 years), sex, and level of education. Before respondents completed the questionnaire, they were informed about the background of the study, the target population of adolescents with problems of substance abuse and/or delinquency, and the attributes and levels used in the choice tasks. Furthermore, an outline of the questionnaire, instructions on the type of questions, and a privacy statement were provided. The questionnaire of the pilot and main study comprised four parts. Part one included questions about demographics of the respondent. Part two consisted of the choice tasks. Part three stated questions about the feasibility and readability of the choice tasks. Part four consisted of questions on current health status of the respondent.

### Discrete choice experiment

DCEs are frequently used to inform policy decisions in health care [29–31]. In such experiments, individuals are confronted with a series of choice tasks. The DCE methodology is based on McFadden’s random utility theory [32] and assumes that the utility of an alternative (here: an ASC T-ASI state) is derived from its characteristics (here: ASC T-ASI domains and levels) and that individuals, when confronted with a choice task that consists of *n* alternatives with a fixed number of attributes and levels, will choose the alternative that maximizes their utility. The utility function for respondent *i* is written as *U*_*i*_ = *V*_*i*_ + *ε*_*i*_ where *V*_*i*_ refers to the systematic component of the utility function which reflects the observed influences of attributes and levels and *ε*_*j*_ to the stochastic component of the utility function which reflects unobserved influences [32].

**Fig. 1:**
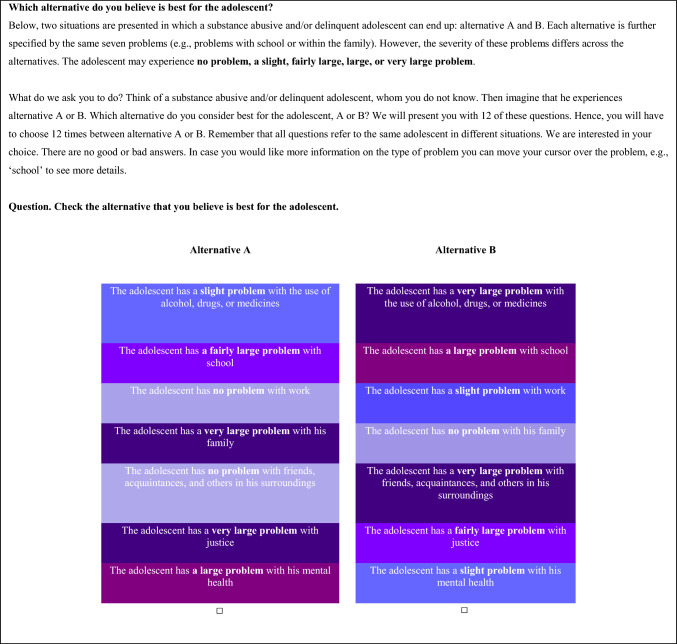
Example choice task. Attributes and levels are presented in white for clarity

***Choice task*** The current study used choice tasks with two unlabeled alternatives (A and B) reflecting a state of an adolescent described by the seven domains (substance use, school, work, family, social relationships, justice, and mental health) and five levels (ranging from ‘no problem’ to ‘very large problem’) of the ASC T-ASI. Respondents were asked to adopt a ‘societal perspective’ and choose the alternative *that they believed would be best for an adolescent at this moment* [33]. This perspective resembles that in the choice tasks applied for obtaining a value set for the EQ-5D instrument for children and adolescents [34]. In this way, we obtained societal preferences for the different situations of the adolescent described with the ASC T-ASI instrument. Figure 1 presents an example of one of the choice tasks, as presented to the respondents.

Between 11 and 16 December 2013, we collected pilot data from a sample (*n* = 106) representative of the general adult population in the Netherlands in terms of age (18–65 years), sex, and education level. The pilot study had two main objectives. First, we collected information on the attributes and levels that could be used for the development of an efficient design for the main study. Second, we obtained information concerning the feasibility and readability of the twelve choice tasks completed by the respondents. Two of the twelve choice tasks concerned control tasks that were included to identify respondents who responded inconsistently. The first task was a dominated choice scenario, with one alternative indicating less problems in all domains. The second control task presented respondents with a mirrored version of a choice task they had already answered earlier on in the DCE. Respondents who answered at least one of the two control questions inconsistently were excluded.

Between 7 and 13 March 2014, we collected main data from a sample (*n* = 1500) representative of the general adult population in the Netherlands in terms of age (18–65 years), sex, and education level. Based on the results of the pilot study, the design of the main study was slightly adapted. The number of choice tasks per respondent was reduced from ten to eight and color-coding was applied to the choice tasks to visually emphasize the differences in problem severity between attribute levels. A D-efficient design with 40 rows and five blocks was created by applying normally distributed Bayesian priors estimated based on the results of the pilot study, using 1000 Halton draws. The attribute levels were dummy-coded due to the uneven spacing between them, which is a characteristic common to Likert scales. Respondents were randomly assigned to one of five blocks with eight choice tasks each plus the two control tasks. Respondents were excluded from the analyses when they ‘answered at least one of the two control questions inconsistently or when they were identified as ‘speeders’. The latter completed the choice tasks in less 4 min (i.e., less than one third of the mean completion time of two independent researchers). Data quality was further improved by including an alternative-specific constant to the regression model, based on which potential left–right bias was assessed.

### Model specification

The main data were analyzed by first applying an MNL model and stepwise extending this model toward a panel mixed multinomial logit (MMNL) model. Whereas for the MNL model the assumption holds that all variables need to be independently and identically distributed (IID assumption), this assumption does not apply to the panel MMNL model [35]. The panel MMNL model hence allows for interdependency of observations (which e.g., may occur when respondents answer several choice tasks) and heterogeneity in respondents’ preferences. Consequently, within the panel MMNL model, utility variation, which would otherwise enter into the error component of the MNL model is explicitly modeled and reflected in the parameter estimates [35, 36]. Model fit was evaluated based on log likelihood ratio (LR) tests.

When extending the MNL model toward a panel MMNL model, several steps were taken. First, an unrestricted dummy-coded MNL model with an alternative-specific constant was estimated. No evidence for left–right bias was found, and hence the constant was excluded from the model. Next, we investigated various model specifications with random parameters to allow for heterogeneity in respondents’ preferences. Making all parameters random was not feasible technically due to insufficient data, so stepwise parameters that indicated the strongest heterogeneity, i.e., with the highest standard error, were added as random parameters and model fit was evaluated based on LR tests. As a final step, we verified whether collapsing attribute levels two and three (‘fairly large problem’ and ‘large problem’) or three and four (‘large problem’ and ‘very large problem’) would improve model fit. These modifications did not lead to an improvement based on LR. Hence, a panel MMNL with fourteen random and fourteen fixed parameters was chosen as the final model. Standard deviations were derived based on Cholesky decomposition. The analyses were performed in NLOGIT (version 5).

### ASC T-ASI preference scores

To estimate the coefficients for the fourteen random parameters, bootstrapping using 10,000 hypothetical individuals from a normal distribution using the population level estimates of the MMNL was applied and individual-specific parameters were derived. Individual-specific parameters for each of the attributes and levels were averaged. The averages of the random parameters and estimates of the beta coefficients of the non-random parameters from the MMNL model were rescaled to a 0–1 scale to provide an ASC T-ASI tariff set. A score of 0 refers to the worst state with very large problems in all of the ASC T-ASI domains, while a score of 1 refers to the best state with no problems in any of the domains.

## Results

The main study comprised 1500 respondents (after the exclusion of respondents who answered at least one of the control questions inconsistently and ‘speeders’). General respondent characteristics are displayed in Table 1.

**Table 1:** Sample characteristics of main study (*n* = 1500)

	Sample main study Mean (SD) or %	General Dutch population^c^ Mean (SD) or %
Gender (male)	50.3	50.2
Age		
Female		
18–34	16.3	16.2
35–49	16.8	16.7
50–65	16.7	16.9
Male		
18–34	16.5	16.5
35–49	16.9	16.8
50–65	16.9	17.0
Educational level^a^		
Low	10.9	31.3
Middle	58.6	39.5
High	30.5	27.2
Unknown	–	2.0
Completion time (min)		
Minimum	4.00	
Maximum	23.70	
Mean	8.16 (3.26)	
Work		
Yes (> = 36 h/week)	29.3	
Yes (< 36 h/week)	27.6	
No	43.1	
Children		
Yes (< 12 years)	13.1	
Yes (12–21 years)	18.5	
Yes (> 21 years)	26.3	
No	50.8	
Subjective health (EQ-5D-3L VAS)	74.35 (14.78)	77.72 (15.19)^b^
Health-related QoL (EQ-5D-3L)	0.87 (0.20)	0.87 (0.18)^b^

^a^Low = lower vocational and primary school, Middle = middle vocational and secondary school, High = higher vocational and academic education

^b^[32]

^c^Age is based on statistics for the population aged 18–75 years, sex is based on statistics for the overall population, and education level is based on statistics for the population aged 15–75 years. Population statistics for 2013; Source: Statistics Netherlands (https://opendata.cbs.nl/statline)

Respondents’ distribution of age and sex was in line with the general adult population in the Netherlands. Mean age was 42 years (general Dutch population: 42 years) and the proportion of male respondents was 50.3% (general Dutch population: 50.2%) [37]. Respondents’ distribution of education level was less in line with the general adult population in the Netherlands, as respondents more frequently had a middle or higher education level. Completion time ranged from 4 min to nearly 24 min, with a mean completion time of 8 min and 13 s. This shorter completing time as compared to the pilot can be explained by the reduction in the number of choice sets and the addition of color-coding. A large proportion of the respondents stated not to have paid work (43.1%), 29.3% worked 36 or more hours a week and 27.6% worked part time with an average of 19.16 h/week (SD 9.063). Slightly more than half of respondents (50.8%) had children, of which 18.5% were between 12 and 21 years (an age group similar to the population that the discrete choice task referred to). Subjective health and health-related quality of life based on the EQ-5D and its Dutch tariffs were comparable to the values of the general Dutch population [38].

### Preference scores for the ASC T-ASI domains and problem levels

The results of the panel MMNL model are presented in Table 2. An overview of the coefficients for each of the attributes and attribute levels is provided.

**Table 2:** Results of panel mixed multinomial logit regression model (main study)

Attribute	Level	B coefficient	Standard deviation
Substance use	No problem	2.48564***	1.63320***
	Slight problem	1.92631***	1.39210***
	Fairly large problem	0.87412***	–
	Large problem	0.33400***	–
	Very large problem	Base	
School	No problem	0.91027***	–
	Slight problem	0.86616***	0.37781***
	Fairly large problem	0.33925**	1.10870***
	Large problem	0.25556***	–
	Very large problem	Base	
Work	No problem	1.25942***	1.08894***
	Slight problem	0.81447***	–
	Fairly large problem	0.58935***	–
	Large problem	0.21731*	0.79275***
	Very large problem	Base	
Family	No problem	1.67697***	–
	Slight problem	1.17190***	–
	Fairly large problem	0.58164***	0.85331***
	Large problem	0.03268	0.94046***
	Very large problem	Base	
Social relationships	No problem	1.26387***	0.73073***
	Slight problem	1.01831***	–
	Fairly large problem	0.78538***	–
	Large problem	0.37928***	1.02409***
	Very large problem	Base	
Justice	No problem	2.02321***	1.53078***
	Slight problem	1.53178***	1.09865***
	Fairly large problem	0.68487***	–
	Large problem	0.25052**	0.66371***
	Very large problem	Base	
Mental health	No problem	2.31869***	1.06997***
	Slight problem	1.95064***	–
	Fairly large problem	1.21795***	–
	Large problem	0.52154***	–
	Very large problem	Base	

***, **, * indicate statistical significance at 1%, 5%, 10% level respectively; – indicate fixed parameters

Table 2 shows that all coefficients were positive, indicating that fewer problems than the base case level (‘very large problems’) were preferred by the respondents. The coefficients of problems with substance use, family, justice, and mental health were relatively large compared to the other coefficients indicating that changes in these domains—and particularly having a ‘large problem’ and a ‘very large problem—had a relatively high impact on respondents’ choices between the alternative ASC T-ASI states. Problems in the domains school, work, and social relationships had a relatively low impact on their choices. All but two coefficients were significant at the 5% level. One coefficient for ‘large problems’ was only significant at the 10% level (with an effect of 0.019 on the tariff), and one was not significant at the 10% level (with a marginal effect of 0.001 on the tariff). This suggests that in these two cases, the level ‘large problem’ was not found to be significantly different from the level ‘very large problem’ (base level). Yet, as described above, collapsing the problem levels ‘large’ and ‘very large’ did not yield an improvement of the model (as shown by the LR).

Table 2 also shows that all standard deviations of the random parameters were relatively large and significant at the 1% level hence providing evidence for preference heterogeneity among respondents.

Table 3 presents the results of the conversion of the coefficients into preference scores per domain and problem level with the total score ranging from 0 to 1. A score of 0 refers to the worst state as described by the ASC T-ASI (i.e., with large problems in all domains) and a score of 1 refers to the best possible state described by the instrument (i.e., no problems in any domain).

**Table 3:** ASC T-ASI tariff set

Domain	Problem level	Preference Scores
Substance use	No problem	0.210
	Slight problem	0.161
	Fairly large problem	0.073
	Large problem	0.028
	Very large problem	0.000
School	No problem	0.076
	Slight problem	0.073
	Fairly large problem	0.028
	Large problem	0.022
	Very large problem	0.000
Work	No problem	0.105
	Slight problem	0.068
	Fairly large problem	0.050
	Large problem	0.019
	Very large problem	0.000
Family	No problem	0.141
	Slight problem	0.098
	Fairly large problem	0.049
	Large problem	0.001
	Very large problem	0.000
Social relationships	No problem	0.106
	Slight problem	0.086
	Fairly large problem	0.066
	Large problem	0.032
	Very large problem	0.000
Justice	No problem	0.168
	Slight problem	0.128
	Fairly large problem	0.058
	Large problem	0.022
	Very large problem	0.000
Mental health	No problem	0.194
	Slight problem	0.164
	Fairly large problem	0.102
	Large problem	0.044
	Very large problem	0.000

The use of the preference scores can be illustrated as follows: Based on Table 3, an adolescent with a ‘slight problem’ in the domain substance use, a ‘fairly large problem’ in the domains school and work and ‘no problem’ in the domains family, social relationships, justice, and mental health would be coded 2,331,111, which translates into a score of 0.161 + 0.028 + 0.050 + 0.141 + 0.106 + 0.168 + 0.194 = 0.848.

Consistent with the coefficients presented in Table 2 and the abovementioned example, it can be seen that the domains substance use, mental health, justice, and family were more influential and received more weight than the domains social relationships, work, and school.

## Discussion

In this study, we obtained societal preference scores for the ASC T-ASI, contributing to the availability of a first, short, preference-based measure suitable for self-completion, and use in economic evaluations of systemic family interventions. Our primary aim was to obtain broad societal preference scores for the ASC T-ASI. We adopted a societal perspective, also given the broad scope of outcomes. The scope of this measure is deemed to be more in line with the goals of systemic interventions than currently available generic health-related quality of life measures, and hence enables a more comprehensive measurement of the effects of such interventions. The need for such measures in the context of substance abuse treatment was noted before [39]. The ASC T-ASI is an adaptation of the frequently used T-ASI [27], which may contribute to its acceptance, validity, and feasibility of implementation [16, 40]. We used a two stage-design, starting with an elaborate pilot study, followed by a large main study. Advantages of this approach were that adjustments to the design could be made in between the pilot and main study, enhancing the quality of the data obtained. We allowed interdependency of observations and heterogeneity in preferences in our analyses and the selected model fitted the panel data of the choice tasks and accounted for individual differences in choice behavior. The performed DCE yielded societal preference scores that showed logical orderings, and the different levels within each domain almost all were statistically significantly different from each other. The results indicated that the domains substance use, mental health, justice, and family were most important in our sample, representative of the Dutch population (aged 18–65 years) in terms of age, sex, and education. With these tariffs, the ASC T-ASI can be seen as a validated [16, 40], preference-based outcome measure with a scoring system ranging from 0 (worst state described with the instrument) and 1 (best state described with the instrument).

Before addressing some implications of this study, and discussing the use of the here derived preference scores, some limitations of this study need noting. First, one may argue that some of the included domains in the ASC T-ASI may not be relevant for all adolescents. For example, the domain ‘work’ may only be relevant for relatively old adolescents who work or would want to work [16]. Future research may investigate this issue further, for instance, by considering conditional questions or changes in the labeling of the domains or levels. Second, two parameters presented in Table 2 (family—‘large problems’ and work—‘large problems’) were not statistically significant at the 5% level. Note that the impact of the non-significant coefficients on the tariff was small (with values of 0.019 and 0.001, respectively). Merging the levels reduced the model fit. Hence, we chose to keep the separate levels. Third, implausible domain combinations and interactions between preferences for the attributes and levels of the ASC T-ASI were not explicitly accounted for in the study design [41]. In this first study deriving preference scores, we first focused on estimating main effects in order to allow establishing an ASC T-ASI tariff set for use in economic evaluations, in line with other tariffs (e.g., for EQ-5D and SF-6D instruments) which are usually additive. Furthermore, evidence on any interactions between preferences for ASC T-ASI attributes and levels was lacking, and accounting for all possible interaction effects—in addition to the main effects—would have resulted in a highly complex design. This, in turn, would potentially have resulted in increased cognitive burden for respondents or have required data collection in even larger pilot and main samples to maintain power, which was not feasible in this study. Interaction effects are important to explore in future research though. Fourth, the choice tasks were complex for respondents. After the pilot study we, therefore, decreased the number of choice tasks from ten to eight per respondent and applied color-coding to simplify the decision process and reduce cognitive demands to respondents. Moreover, respondents who answered the control questions inconsistently and ‘speeders’ were excluded from the analyses. Nonetheless, in the main study, a majority of the included respondents still considered the choice task to be (very) difficult. In total, 44.1% (*n* = 661) of respondents considered making a choice between the different states to be difficult, which may reflect the inherently difficult nature of the presented choices—also in relation to the Likert scale on which the attribute levels were presented in the choice tasks. Although we accounted for the uneven spacing between the levels (by means of dummy coding) in the analyses, we do not know to what extent respondents took this into account when making their choices and how this may have influenced our results [42]. The study design did not include an opt-out option [43], which may also have increased the difficulty and influenced our results. Fifth, potentially related to the previous point, we excluded a substantial number of respondents who answered one or both of the two control questions inconsistently. This was a strict rule, imposed in order to achieve the highest possible quality of data for the tariff set. Respondents who were excluded due to answering one control question inconsistently (*n* = 717) were significantly older (44.91 vs. 42.00 years; *p* = 0.000) and lower educated (*p* = 0.000) than included respondents. No difference in sex was observed. Sixth, duration of the ASC T-ASI states was not included as an attribute in the DCE, nor were additional time trade-off tasks used to anchor the tariff set on a ‘natural 0’. The latter means, like in other outcome measures like, e.g., the ICECAP [5, 6], the zero in in the obtained ASC T-ASI tariff does not equal the state of ‘dead’ but to the worst state defined by the scale. This also implies a different interpretation of changes on the instrument than in case of conventional QALY measures, as elaborated on below. The former, i.e., not specifying duration, implies that we could not observe discounting or duration effects in our study. Seventh, the current study was limited to the Dutch setting. Moreover, our sample was representative for the Dutch general population in terms of age and sex, but less so for education level. Furthermore, among our respondents, there was a high percentage of individuals without paid work. Respondents’ socio-demographic characteristics may have influenced our results. Future research may consider assessing the direction and size of this potential impact. Eighth, we observed quite some preference heterogeneity in the DCE. While the aim of the current study was to obtain overall preference scores rather than to differentiate between the scores of specific groups of respondents, it may be worth exploring this further in future studies.

Next to these limitations, the meaning and the interpretation of the here derived scores are distinct from those related to common HRQOL instruments for several reasons. First, conceptually, QALYs intend to measure *health-related* quality of life, whereas the ASC T-ASI aims to measure broader, and arguably less well-defined, ‘*addiction-related* quality of life’ in adolescents. This means it intends to capture a different concept and hence cannot be readily compared to or combined with QALY measures. Second, as already mentioned above, for generic health-related quality of life measures, like the EQ-5D, a preference-score or utility of 0 corresponds to the state ‘dead’ and hence represents a ‘natural zero’. This is not the case for the ASC T-ASI, where a score of 0 simply refers to the most severe problems in all domains of the instrument. This state could, in the more general sense of the word utility, still be associated with positive or negative utility. Combining ASC T-ASI scores with duration therefore requires a careful consideration and interpretation. This is similar to other recently developed broader outcome measures, like the ICECAP instruments [5, 6]. Third, QALY tariffs typically represent average valuations of health states obtained by asking respondents to imagine being in these health states themselves. Here, we asked adults to value states from a societal perspective, i.e., *for* an adolescent, which leads to fundamental differences. The observed scores reflect what people in the general public think is ‘best’ for the adolescent involved, rather than an indication of a preference to be in a certain state oneself. Hence, even the word ‘preference’ should be interpreted and understood in that context. This represents a crucial difference with other tariffs and common ‘utility scores’, which needs strong emphasis. It also emphasizes that the scores obtained here cannot be straightforward compared to, let alone aggregated with, QALYs. The approach adopted in this study shows similarities with valuation approaches in the context of child health (e.g., the valuation protocol of EQ-5D-Y-3L [34]). We requested (adult) respondents to select the better state ‘for the adolescent’, resulting in preferences that may be seen as somewhat ‘paternalistic’. Such preferences may be preferred over those of adolescents themselves (especially those experiencing these states), if one believes these preferences may be 'distorted' by underlying issues, such as alcohol and drug problems, or ill-informed or myopic due the age of adolescents. Moreover, such preferences could be influenced by mechanisms of coping and adaptation [44]. Nonetheless, future research on how the outcomes and the response patterns of valuation would differ when taking alternative perspectives or using alternative sources of valuation remains warranted, like recently done for child health [45]. Finally, we would also like to note that the respondents’ preferences may also be influenced by other elements not included in the ASC-T-ASI, such as the adolescent’s age and family circumstances. It is unclear whether respondents missed such information, whether they assumed a specific context, and whether the influence of such items would be significant. Future research could explore the potential influence of any systematic preferences that may be associated with ASC T-ASI states (beyond those directly related to its domains and levels) on the tariff set, and the potential implications this may have for policy.

Given the above, it is good to highlight that we intentionally opted for this valuation approach in the current context for several reasons. We set out to obtain societal preferences from the general population, in line with Dutch guidelines for economic evaluations in health care [1]. Given that the ASC T-ASI relates to adolescents, this implied, for almost all respondents, valuing not only hypothetical states but also for a person with different age and context than those of the respondents. This required additional instructions, as we did not want to obtain general preferences for ‘states of substance abuse’ but specifically in relation to the phase of adolescence. We framed the choices in terms of the best state for the adolescent, which may lead to somewhat ‘paternalistic’ (rather than more hedonistic) choices. This was done to stay close to the purpose of many interventions in this area. All these choices are inherently normative, and it is interesting to further investigate them and their influence on preferences in future studies. For instance, an alternative would have been to use preferences of adolescents actually being in these states. Besides practical issues of recruiting these adolescents, also normative issues regarding whether their preferences (including, for example, those related to substance abuse or school performance) would be most useful for societal decision-making. One could argue such preferences of adolescents actually experiencing these states could be influenced by coping and adaptation [45], but also by underlying problems like addiction and myopia (also due to the age of respondents). Other sources, like adolescents not experiencing these states and issues, or the general public like used in this study, all come with own limitations.

Future research could explore the important normative issue of ‘whose values count’ [44] in situations like these, but could also compare preferences of affected adolescents, adolescents without the specific problems described with the instrument, and those of the general public. Using preferences from non-affected adolescents may yield preferences that are more representative of those of the treated group. Moreover, arguably, such respondents might be more capable of imagining (what it means) being in the different states described with the ASC T-ASI than adults in the general population. However, whether their preferences would be (more) appropriate to use in societal decision-making remains unclear.

Future research may also consider the framing of the choice task. We chose the framing of asking which situation was ‘best for the adolescent’, reflecting potential treatment goals of the health system, which can be different from what the adolescent would prefer. The approach taken can, therefore, be viewed as being aligned with societal decision-making and collective financing of interventions, but this may come at the expense of not using (current or future) preferences of the treated adolescents.

In combination, these differences mark a fundamental distinction between the instrument presented here and the common HRQOL instruments. This also implies that using the ASC T-ASI leads to incomparability with conventional CUAs. Nonetheless, the ASC T-ASI can be used instead of, or (preferably at this stage) in addition, to generic HRQOL instruments as its use may be more informative and appropriate when performing economic evaluations of systemic family interventions where effects broader than health are expected. It also facilitates comparisons of benefits across such interventions. Indeed, the ASC T-ASI and the here presented tariffs can be used in several ways. It may be used as an add-on instrument in future cost-effectiveness studies and clinical trials with low burden to patients due to its brevity. Also, it can be used as a stand-alone self-completion instrument to weight changes in the situation of adolescents in the captured domains. Both options would provide valuable information for use in economic evaluations. When the ASC T-ASI is used in combination with other cost or benefit measures in economic evaluation, overlap and double counting need to be avoided. Such overlap could occur with common measures like the EQ-5D [16] or with cost components of economic evaluations. This, as well as the validity of the ASC T-ASI in different settings, needs to be investigated further in future research [16, 40]. Furthermore, though the ASC T-ASI is developed in the context of systemic family interventions, future studies may consider its application in a broader context of youth mental health interventions.

In conclusion, we performed a DCE to obtain societal preference scores for the ASC T-ASI facilitating its use in the context of economic evaluations of systemic family interventions in adolescents with problems with substance use and/or delinquency. To our knowledge, the ASC T-ASI is the first preference-based measure in adolescent mental health care for which societal preference scores have been obtained that capture benefits beyond those included in the QALY. As such, the results of this study may contribute to better reflecting of the value for money offered by such interventions and optimize decisions on their reimbursement. Many questions for further research were identified which exceed the scope of the current study. Nonetheless, the presented tariff may provide a first step in including relevant disease-specific aspects in economic evaluations of systemic family interventions.

### Supplementary Information

Below is the link to the electronic supplementary material.

Supplementary file1 (DOCX 23 KB)

## Data Availability

Data supporting this study are available upon request from the corresponding author at hakkaart@eshpm.eur.nl.
