# Supplementary material for: Obtaining preference scores for an abbreviated self-completion version of the Teen-Addiction Severity Index (ASC T-ASI) to value therapy outcomes of systemic family interventions: a discrete choice experiment
Source: Eur J Health Econ. 2023 Sep 27;25(5):903–13. doi: 10.1007/s10198-023-01633-3 (PMC11192667; doi:10.1007/s10198-023-01633-3)
Supplement: Supplementary file 1 — Supplementary file1 (DOCX 23 KB) [file 10198_2023_1633_MOESM1_ESM.docx]

## Appendix A. English version of the ASC T-ASI instrument (B1 level) [16]

Please check the answer that currently fits you best:

1. **Substance use**

I have **no problem** with the use of alcohol, drugs or medicine ❑

I have a **slight problem** with the use of alcohol, drugs or medicine ❑

I have a **fairly large problem** with the use of alcohol, drugs or medicine ❑

I have a **large problem** with the use of alcohol, drugs or medicine ❑

I have a **very large problem** with the use of alcohol, drugs or medicine ❑

1. **School**

I have **no problem** with school ❑

I have a **slight problem** with school ❑

I have a **fairly large problem** with school ❑

I have a **large** **problem** with school ❑

I have a **very large problem** with school ❑

1. **Work**

I have **no problem** with work ❑

I have a **slight problem** with work ❑

I have a **fairly large problem** with work ❑

I have a **large problem** with work ❑

I have a **very large problem** with work ❑

1. **Family**

I have **no problem** with family ❑

I have a **slight problem** with family ❑

I have a **fairly large problem** with family ❑

I have a **large problem** with family ❑

I have a **very large problem** with family ❑

1. **Social relationships**

I have **no problem** with friends, acquaintances and others in my environment ❑

I have a **slight problem** with friends, acquaintances and others in my environment ❑

I have a **fairly large problem** with friends, acquaintances and others in my environment ❑

I have a **large problem** with friends, acquaintances and others in my environment ❑

I have a **very large problem** with friends, acquaintances and others in my environment ❑

1. **Justice**

I have **no problem** with the judicial authorities ❑

I have a **slight problem** with the judicial authorities ❑

I have a **fairly large problem** with the judicial authorities ❑

I have a **large problem** with the judicial authorities ❑

I have a **very large problem** with the judicial authorities ❑

1. **Mental health**

I have **no problem** with my mental health ❑

I have a **slight problem** with my mental health ❑

I have a **fairly large problem** with my mental health ❑

I have a **large problem** with my mental health ❑

I have **a very large problem** with my mental health ❑

## Appendix B. Dutch version of the ASC T-ASI instrument (B1 level) [24]

Zet één kruisje bij het antwoord dat op dit moment het best bij jou past:

1. **Middelengebruik**

Ik heb **geen probleem** met het gebruik van alcohol, drugs of medicijnen ❑

Ik heb een **klein probleem** met het gebruik van alcohol, drugs of medicijnen ❑

Ik heb een **redelijk groot probleem** met het gebruik van alcohol, drugs of medicijnen ❑

Ik heb een **groot probleem** met het gebruik van alcohol, drugs of medicijnen ❑

Ik heb een **heel groot probleem** met het gebruik van alcohol, drugs of medicijnen ❑

1. **School**

Ik heb **geen probleem** met school ❑

Ik heb een **klein probleem** met school ❑

Ik heb een **redelijk groot probleem** met school ❑

Ik heb een **groot probleem** met school ❑

Ik heb een **heel groot probleem** met school ❑

1. **Werk**

Ik heb **geen probleem** met werk ❑

Ik heb een **klein probleem** met werk ❑

Ik heb een **redelijk groot probleem** met werk ❑

Ik heb een **groot probleem** met werk ❑

Ik heb een **heel groot probleem** met werk ❑

1. **Familie**

Ik heb **geen probleem** met familie ❑

Ik heb een **klein probleem** met familie ❑

Ik heb een **redelijk groot probleem** met familie ❑

Ik heb een **groot probleem** met familie ❑

Ik heb een **heel groot probleem** met familie ❑

1. **Sociale relaties**

Ik heb **geen probleem** met vrienden, bekenden en anderen in mijn omgeving ❑

Ik heb een **klein probleem** met vrienden, bekenden en anderen in mijn omgeving ❑

Ik heb een **redelijk probleem** met vrienden, bekenden en anderen in mijn omgeving ❑

Ik heb een **groot probleem** met vrienden, bekenden en anderen in mijn omgeving ❑

Ik heb een **heel groot probleem** met vrienden, bekenden en anderen in mijn omgeving ❑

1. **Justitie**

Ik heb **geen probleem** met justitie ❑

Ik heb een **klein probleem** met justitie ❑

Ik heb een **redelijk groot probleem** met justitie ❑

Ik heb een **groot probleem** met justitie ❑

Ik heb een **heel groot probleem** met justitie ❑

1. **Geestelijke gezondheid**

Ik heb **geen probleem** met mijn geestelijke gezondheid ❑

Ik heb een **klein probleem** met mijn geestelijke gezondheid ❑

Ik heb een **redelijk groot probleem** met mijn geestelijke gezondheid ❑

Ik heb een **groot probleem** met mijn geestelijke gezondheid ❑

Ik heb een **heel groot probleem** met mijn geestelijke gezondheid ❑
